# **Retraction notice for:** Notoginsenoside R1 upregulates miR-221-3p expression to alleviate ox-LDL-induced apoptosis, inflammation, and oxidative stress by inhibiting the TLR4/NF-κB pathway in HUVECs

**DOI:** 10.1590/1414-431X2024e9346retraction

**Published:** 2025-01-31

Lingbo Zhu^1*^ https://orcid.org/0000-0002-6498-9562, Xinyan Gong^1*^ https://orcid.org/0000-0002-2148-1566, Jianping Gong^1^ https://orcid.org/0000-0003-0045-4638, Yungang Xuan^1^ https://orcid.org/0000-0003-2486-1764, Ting Fu^1^ https://orcid.org/0000-0003-3518-6469, Shimao Ni^1^ https://orcid.org/0000-0003-0981-3713, Lei Xu^1^ https://orcid.org/0000-0002-4385-2807, and Ningning Ji^1^ https://orcid.org/0000-0003-0478-3447

^1^Department of Cardiology, Central Hospital of Yiwu, Yiwu, Zhejiang, China

Correspondence: Ningning Ji: <Ji_Ningning78@outlook.com>

*These authors contributed equally to this study.

Retraction notice for: Braz J Med Biol Res (2020) 53(6): 9346 | doi: 10.1590/1414-431X20209346 | PMID: 32401923 | PMCID: PMC7233198

The authors requested retraction of the article “Notoginsenoside R1 upregulates miR-221-3p expression to alleviate ox-LDL-induced apoptosis, inflammation, and oxidative stress by inhibiting the TLR4/NF-κB pathway in HUVECs” that was published in volume 53, no. 6 (2020) (Epub May 8, 2020) in the Brazilian Journal of Medical and Biological Research.

The author Lingbo Zhu stated that “PubPeer questioned the authenticity of the results of this article and comparison of the figures with other publications that had been retracted. Due to the age of the data and the lack of access to the original data, the authors request that the article be retracted”.

The Editors decided to retract this paper to avoid further damage to the scientific community. The Brazilian Journal of Medical and Biological Research remains vigilant to prevent misconduct and reinforces the Journal’s commitment to good scientific practices.

Editorial Team - Brazilian Journal of Medical and Biological Research

